# Mitochondrial DNA copy number associated dementia risk by somatic mutations and frailty

**DOI:** 10.1007/s11357-024-01355-1

**Published:** 2024-09-23

**Authors:** Qu Tian, David A. Zweibaum, Yong Qian, Richard F. Oppong, Luke C. Pilling, Francesco Casanova, Janice L. Atkins, David Melzer, Jun Ding, Luigi Ferrucci

**Affiliations:** 1 Translational Gerontology Branch, National Institute on Aging Intramural Research Program, 251 Bayview Blvd., Suite 100, Baltimore, MD 21224 USA; 2 Epidemiology & Public Health Group, Department of Clinical & Biomedical Science, Faculty of Health & Life Sciences, University of Exeter, College House, University of Exeter, St Luke’s Campus, Heavitree Road, Exeter Devon, EX1 2LU UK

**Keywords:** Mitochondria, DNA copy number, Human blood, Dementia, Frailty, Somatic mutation

## Abstract

Mitochondrial dysfunction is linked to physical impairment and dementia. Mitochondrial DNA copy number (mtDNAcn) from blood may predict cognitive decline and dementia risk, but the effect of somatic mutations or frailty is unknown. We estimated mtDNAcn using *fastMitoCalc* and microheteroplasmies using *mitoCaller*, from Whole Genome Sequencing (WGS) data. In 189,566 participants free of dementia at study entry (mean age = 56 ± 8), we examined the association between mtDNAcn and subsequent dementia diagnosis using Cox regression. Cognition was assessed in a subset on average 8.9 years later. We examined the associations between mtDNAcn and cognitive measures using multivariable linear regression, adjusted for demographic factors, mtDNAcn-related parameters, and *apolipoprotein E ε4* status. We further stratified by frailty and microheteroplasmies. Over an average follow-up of 13.2 years, 3533 participants developed dementia. Each SD higher mtDNAcn (16) was associated with 4.2% lower all-cause dementia hazard (HR = 0.958, *p* = 0.030), 6% lower non-AD dementia hazard (HR = 0.933, *p* = 0.022), and not-AD dementia hazard. The associations between mtDNAcn and all-cause dementia and non-AD dementia were stronger among those who were pre-frail or frail or with higher microheteroplasmies. Higher mtDNAcn was associated with higher DSST scores (*p* = 0.036) and significant only among those with higher microheteroplasmies or frailty (*p* = 0.029 and 0.048, respectively). mtDNAcn was also associated with delta TMT and paired associate learning only in pre-frail/frail participants (*p* = 0.007 and 0.045, respectively). Higher WGS-based mtDNAcn in human blood is associated with lower dementia risk, specifically non-AD dementia, and specific cognitive function. The relationships appear stronger in high somatic mutations or frailty. Future studies are warranted to investigate biological underpinnings.

**Supplementary Information:**

The online version contains supplementary material available at 10.1007/s11357-024-01355-1.

## Background

Age-related mobility decline predicts mild cognitive impairment, a transitional stage from normal brain aging to Alzheimer’s disease (AD) dementia, up to 12 years before the symptom onset [1]. Recent studies have shown that dual cognitive and mobility decline is associated with a higher dementia risk than cognitive decline or mobility decline only [2, 3]. These data are in line with the evidence that people with the presence of physical frailty had a higher prevalence of AD than those without frailty even at a low amount of AD pathology [4].

Mitochondrial dysfunction, a hallmark of aging and frailty, has been proposed to contribute to the pathogenesis of AD, either directly or indirectly by triggering neuroinflammation [5]. Lower mitochondrial function has been associated with mobility decline in older adults and amyloid precipitation in human cell culture studies. Mitochondrial dysfunction may be a shared pathway between cognitive and mobility decline that eventually leads to dementia [6, 7]. In animal models, mitochondrial dysfunction causes excessive reactive oxygen species (ROS) production and triggers a pro-inflammatory response that can contribute to amyloid formation and neurodegeneration [5]. In humans, mitochondrial oxidative phosphorylation (OXPHOS) produces most of the energy required for diverse biological functions including fueling the resilience mechanisms that contrast damage accumulation and allow biogenesis and repair. Examples of these mechanisms are DNA repair, proteostasis, and mitophagy, all of which have been linked to neurodegeneration and dementia [5]. Thus, mitochondrial function is critical in conditions exhibiting rapid damage accumulation, such as frailty, and it is especially important in energetically demanding tissues such as the brain and muscle.

Assessing mitochondrial function in large epidemiological studies is challenging. However, the blood-based mitochondrial DNA copy number (mtDNAcn), a promising biomarker of mitochondrial function and a proxy for mitochondrial mass, has been successfully employed in epidemiological research. Previous studies of humans have found that mtDNAcn in the blood decreases with advancing age [8], and lower mtDNAcn in the blood is associated with dementia risk and cognition [9, 10]. Although it is unclear whether mtDNAcn in the blood is related to mtDNAcn in the brain, mtDNAcn from human blood and brain samples are both associated with dementia risk or brain pathologies of iron, tau, or TAR DNA-binding protein 43 (TDP-43) [10–12]. However, these previous studies did not examine whether the degree of biological damage affects the role of mitochondrial function in maintaining brain health. We hypothesize that the relationship between mitochondrial function and brain health may be stronger in those individuals with a high degree of biological cell damage operationalized as frailty of high heteroplasmy load because expanding mitochondrial volume may have compensatory effects by supporting resilience strategies [13].

Frailty has been defined as a state of clinical vulnerability, eroded homeostatic reserve, and consequent impairment of multiple physiological systems that implies a high risk of adverse health outcomes. In keeping with this view, previous studies demonstrated that the presence of frailty strongly increases the risk of developing dementia [14]. Heteroplasmies express the degree of mixture of normal and mutated mtDNA molecules in cells. While some of these mutations are maternally inherited, others are somatic mutations resulting from unrepaired DNA damage. mtDNA heteroplasmy load increases with aging, and patients with AD showed higher mtDNA heteroplasmies compared to controls [15]. Induced mtDNA heteroplasmies in mice were associated with multiple pathological manifestations including frailty [16]. In older adults, heteroplasmic mutations and rearrangements may indicate cell damage and are found in tissues with high energetic demands, including skeletal muscle, heart, and brain [17, 18]. mtDNA mutations with a frequency lower than 5% are thought to indicate somatic mutations, referred to as microheteroplasmy load [19, 20].

We tested the hypotheses that mitochondrial mass, operationalized as mtDNAcn in circulating cells, would predict the development of dementia and would be associated with cognition. We hypothesized that this association would be particularly strong in the presence of biological damage. We used a clinical measure of frailty as well as microheteroplasmies as proxy measures of the rate of cellular damage accumulation.

## Methods

### Study sample

Participants are from the UK Biobank, a community-based cohort of 502,387 individuals aged 37 to 73 at study entry between 2006 and 2010. Data on clinical diagnosis of diseases, including dementia, are available for all participants from hospital episode statistics (i.e., inpatient records) up to September 30, 2021. In addition, 45% of the participants also had information from medical records by primary care physicians. Up to January 19, 2023, a subset of 200,009 participants had available data on Whole Genome Sequencing (WGS) from blood samples at the study entry. The source of mtDNA is WGS data from buffy coat samples in the blood. Out of 200,009, 199,817 participants were free of dementia at study entry. A total of 189,509 who had all covariates of interest were included in this analysis. Data used in this study were collected between 2006 and September 2021. The collection and use of UK Biobank data are approved by the Northwest Multi-Center Research Ethics Committee (Research Ethics Committee reference 11/NW/0382). All participants provided informed consent to use their data, health records, and biological materials for research purposes. This research has been conducted using the UK Biobank Resource under Application Number 83534.

### Mitochondrial DNA copy number

Mitochondrial DNA copy number (mtDNAcn) was estimated using the *fastMitoCalc* algorithm on blood-derived WGS data. In brief, the mtDNAcn estimation considers that mitochondrial DNA and autosomal DNA are sequenced with comparable intrinsic efficiency [21]. Therefore, mtDNAcn is proportional to the autosomal DNA copy number, and there are two copies of autosomal DNA in a cell. The following formula was used: *mtDNAcn* = (mtDNA average sequencing coverage/autosomal DNA average sequencing coverage) * 2.

The *fastMitoCalc* algorithm employed in this study randomly selects 0.1% of the nuclear genome to estimate autosomal DNA average sequencing coverage. This approach allows the computation of mtDNAcn to be more than 100 times faster than using complete nuclear genome sequences, while still maintaining a high degree of accuracy (*R*^2^ = 0.998) [16].

### Mitochondrial DNA heteroplasmy and microheteroplasmy load

Heteroplasmy is defined as a mixture of more than one allele at a position in mtDNA within a cell. We identified mtDNA heteroplasmies of each study participant by analyzing their WGS data with *MitoCaller* [8], which implements a likelihood-based model to identify mtDNA variants. The heteroplasmy load, defined as the aggregate number of heteroplasmies on the genome, was estimated for each participant. For an mtDNA variant to be classified as a heteroplasmy, it must meet two criteria: (1) all alleles of the variant must be observed in both forward and reverse strand sequence reads, and (2) the minor allele fraction (MAF) for an individual must be at least 3%, a threshold used to prevent false identification of heteroplasmies due to sequencing errors and commonly employed in various studies [22].

As microheteroplasmies (low frequency heteroplasmy) are more likely to be somatic mutations and considered a better predictor than total heteroplasmy, we focused on the effect of microheteroplasmies in this study [19, 20]. We counted microheteroplasmies with a minor allele fraction in the range of 2–5%. Microheteroplasmies that were identified at any of 11 bases (300–302; 309–310; 315–316; 3106–3107; 16,181–16,182) were removed from the heteroplasmy or microheteroplasmy load calculation as these regions are known to be artifact prone [23]. Additionally, six participants with contaminated WGS files, as determined by the presence of a freemix percentage greater than 2%, were removed from analysis [23].

### Frailty

Frailty was defined using the Fried criteria developed in the Cardiovascular Health Study [24], including weight loss, exhaustion, slow walking pace, low physical activity, and low muscle strength. Weight loss, exhaustion, walking pace, and physical activity were measured by self-report. Grip strength was measured using a hydraulic hand dynamometer. Participants who met one to three of these criteria were considered “pre-frail,” and participants who met three or more were considered “frail.” For all self-reported measures, responses of “do not know” and “prefer not to answer” were excluded from all analyses [25].

### Dementia diagnosis

Dementia diagnoses were ascertained from Hospital Episode Statistics (HES) and were available for the whole cohort, from study entry up till September 2021. HES diagnosis data were recorded as ICD-9 or ICD-10 codes. Primary care (General Practice, GP) data were available in 45% of the cohort, up till 2016 or 2017 depending on the data provider (https://biobank.ndph.ox.ac.uk/ukb/label.cgi?id=3000). GP diagnoses were recorded as Read v2 or CTV3 codes. Specific diagnostic codes for dementia were identified from the UK NHS National Institute for Health and Care Excellence (NICE) Quality and Outcomes Framework (QOF) Business Rules (https://qof.digital.nhs.uk), version 37.0 (version date 09/06/2017). Read v2 and CTV3 codes were converted to ICD-9 and ICD-10 codes using UK Biobank Resource 592 (Clinical coding classification systems and maps). The exact codes used are listed in Supplementary Table 1 (Supplementary Table 1). In this study, we examined all-cause dementia, Alzheimer’s Disease (AD), and non-AD dementia. AD dementia was determined based on records from both HES and GP. Non-AD dementia was defined as any dementia diagnosis excluding participants who received a diagnostic code related to AD. For those who developed dementia, time to event was the interval in years from study entry to the date of diagnosis. For those who were free of dementia, time to event was the interval in years from study entry to September 2022 for those who did not have a death record and date of death for those who were deceased. Participants who developed dementia before study entry were excluded from all analyses.

### Cognitive function

Cognitive function was assessed in a subset of participants on average 8.9 years after study entry, including processing speed via Digit Symbol Substitution Test (DSST) (*n* = 13,938), attention via Trail Making Test (TMT) part A (*n* = 13,532), executive function via delta TMT (*n* = 13,426) and tower rearranging task (*n* = 13,817), memory via paired associate learning task (*n* = 14,092), and fluid reasoning via matrix pattern completion task (*n* = 13,933).

### Statistical analysis

We first examined differences in participants’ characteristics by frailty status. We used independent *t* tests for continuous variables or chi-square tests for categorical variables as appropriate. To compare differences in mtDNAcn and microheteroplasmies by frailty status, we adjusted for age using linear regression.

We then examined the associations between mtDNAcn and the incidence of all-cause dementia as well as AD and non-AD dementia using Cox proportional hazards regression. To further understand the effect of frailty and somatic mutation burden, we further performed stratified analysis. All models were adjusted for demographic variables (i.e., baseline age, age-square, sex, race and ethnicity, education, assessment center), smoking status, *APOE ε4* carrier status, and autosomal coverage. Due to a relatively wide age range at the time of mtDNAcn assessment (i.e., the exposure), we added an age-square term if it was significant. To minimize the collinearity of white blood cell-related covariates (i.e., white blood cell count, platelet count, and monocyte, neutrophil, basophil, eosinophil, lymphocyte percentages), we used the R step function to choose covariates that showed the best model fit [26]. To understand the robustness of these associations, we performed several sets of sensitivity analyses. First, we repeated analyses in those aged 60 or older. Second, we additionally adjusted for comorbidities at the time of mtDNAcn assessment. For the stratified analysis by microheteroplasmies, we additionally adjusted for self-reported physical activity.

In the cognitive subset, we further examined the associations between mtDNAcn and each cognitive outcome using multivariable linear regression, adjusted for demographic factors, smoking status, follow-up time, measures related to mtDNAcn assessment, and Apolipoprotein E ε4 status. We then stratified the analysis by frailty status and levels of microheteroplasmy load.

Significance was set at two-sided *p* < 0.05. All statistical analyses were performed in R (version 4.2.1).

## Results

Participants’ characteristics are presented in Table 1. In this sample, 47.6% were classified as pre-frail or frail, and 52.4% were not frail. Compared to the non-frail participants, pre-frail/frail participants were older, more likely to be women and non-white, less likely to have A level or above education, and had a lower percentage of *APOE ε4* carriers (Table 1). The pre-frail or frail participants had relatively shorter follow-up time, higher incidence of all-cause dementia and non-AD dementia, and lower incidence of AD dementia. After adjustment for age, the pre-frail or frail participants showed lower mtDNAcn and higher microheteroplasmies than the non-frail participants (Table 1).

**Table 1:** Participants’ characteristics

	Overall sample (*n* = 189,566)	Non-frail (*n* = 99,383)	Pre-frail/frail (*n* = 90,182)	*p* value
	Mean (SD) or *N* (%)			
Age, years	56.5 (8.1)	56.2 (8.1)	56.7 (8.1)	< 0.001
Women	104,255 (55.0)	53,188 (53.5)	51,067 (56.6)	< 0.001
Race and ethnicity				< 0.001
White	179,561 (94.7)	95,582 (96.2)	83,979 (93.1)	.
Black	2734 (1.4)	1093 (1.1)	1640 (1.8)	.
Asian	3742 (2.0)	1177 (1.2)	2565 (2.8)	.
Others	3529 (1.9)	1531 (1.5)	1998 (2.2)	.
Education				< 0.001
O level or below	63,192 (33.3)	30,056 (30.2)	33,135 (36.7)	.
A level or above	126,374 (66.7)	69,327 (69.8)	57,047 (63.3)	.
Mitochondrial DNA copy number	64.7 (16.0)	65.3 (16.1)	64.1 (15.9)	< 0.001
Microheteroplasmy load	14.7 (10.8)	14.5 (10.7)	14.8 (10.8)	< 0.001
All-cause dementia				
Number of incident cases	3533 (1.9)	1439 (1.4)	2094 (2.3)	< 0.001
Per 1000 person-years	1.41	1.09	1.78	.
AD dementia				
Number of incident cases	1598 (45.2)	735 (51.1)	863 (41.2)	0.001
Per 1000 person-years	0.64	0.56	0.74	.
Non-AD dementia				
Number of incident cases	1935 (54.8)	704 (48.9)	1231 (58.8)	< 0.001
Per 1000 person-years	0.78	0.53	1.05	.
Follow-up time, years	13.2 (2.0)	13.3 (1.8)	13.1 (2.2)	< 0.001
*Apolipoprotein E Ɛ4* carriers	53,673 (28.3)	28,352 (28.5)	25,321 (28.1)	0.030

Notes: O Level: ordinary level. A Level: advanced level. *p* values indicate differences between frail/pre-frail and non-frail participants. *p* values are based on independent *t* tests for continuous variables or chi-square tests for categorical variables

### mtDNA copy number and dementia risk

Over an average follow-up of 13.2 years, 3533 (2%) participants developed dementia, including 1598 AD dementia and 1935 non-AD dementia. In the overall sample after covariate adjustment, each standard deviation higher mtDNAcn (i.e., 16 copies) was associated with a 4.2% lower hazard of all-cause dementia (Table 2). For dementia subtypes, mtDNAcn was associated with non-AD dementia, but not with AD dementia (Table 2). Each standard deviation higher in mtDNAcn was associated with a 6% lower hazard of non-AD dementia.

**Table 2:** The association between mitochondrial DNA copy number and dementia risk stratified by frailty status and microheteroplasmy load

	Overall sample	Stratify by frailty status		Stratify by microheteroplasmies	
		Non-frail	Pre-frail/frail	Lower levels	Higher levels
	HR (95% CI), *p* value				
All-cause dementia	0.958 (0.922, 0.996) 0.030	1.00 (0.947, 1.07) 0.872	0.929 (0.883, 0.978) 0.005	0.973 (0.924, 1.02) 0.287	0.945 (0.886, 1.01) 0.081
AD dementia	0.977 (0.923, 1.03) 0.425	1.04 (0.960, 1.13) 0.329	0.924 (0.853, 1.00) 0.053	0.943 (0.872, 1.02) 0.139	1.04 (0.948, 1.14) 0.406
Non-AD dementia	0.940 (0.892, 0.991) 0.022	0.967 (0.886, 1.05) 0.450	0.928 (0.868, 0.992) 0.028	0.991 (0.925, 1.06) 0.788	0.867 (0.794, 0.947) 0.002

Notes: mtDNAcn and microheteroplasmy load were standardized to *z* scores. All models were adjusted for age, sex, apolipoprotein E ɛ4 carrier status, lymphocyte percentage, sequencing coverage of the autosomal genome, leukocyte count, smoking status, education, and ethnicity. Microheteroplasmies are split into lower and higher levels by a median value

**Fig. 1:**
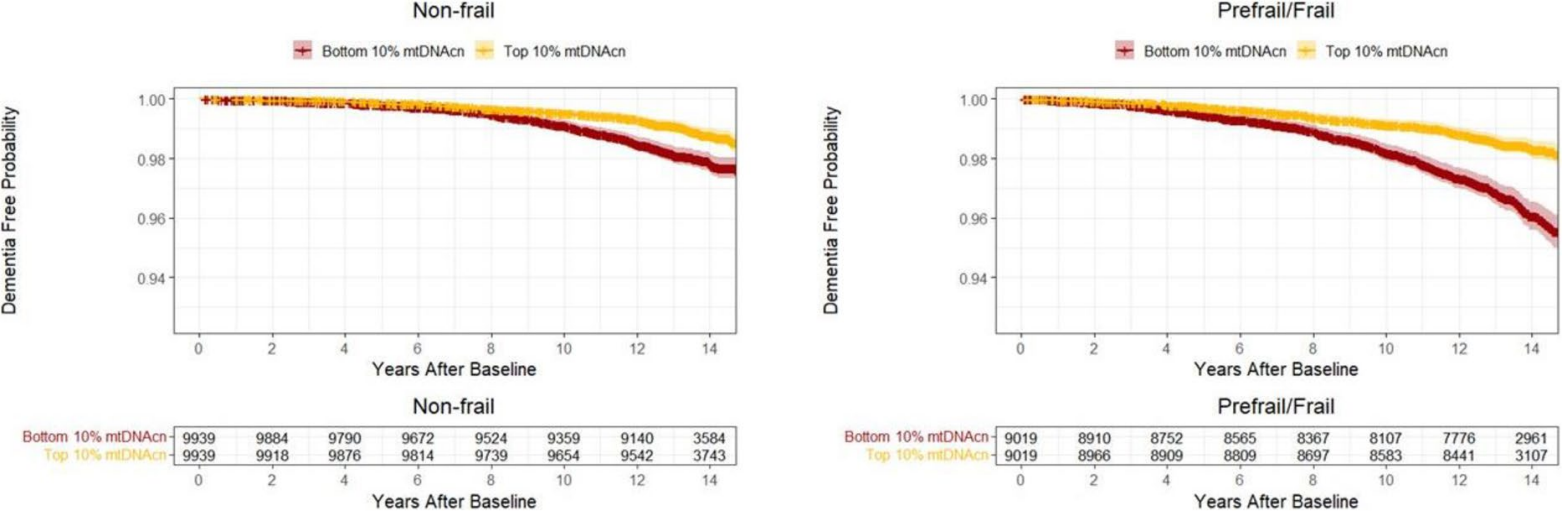
The probability of being free from dementia in the top and bottom 10% of mtDNAcn among participants who were non-frail (left) and prefrail or frail (right). Legend: The median values of mtDNAcn in the top and bottom groups are 94.5 (IQR = 14.9) and 44.2 (IQR = 5.2) among non-frail participants, and 92.6 (IQR = 14.7) and 43.2 (IQR = 4.9) among frail/pre-frail participants, respectively. Reproduced by kind permission of UK Biobank ©

**Fig. 2:**
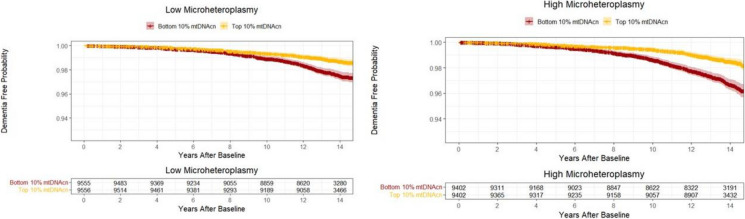
The probability of being free from dementia in the top and bottom 10% of mtDNAcn among participants with low (left) and high (right) microheteroplasmy load. Legend: The median values of mtDNAcn in the top and bottom groups are 101.5 (IQR = 16.6) and 47.8 (IQR = 5.5) among participants with low microheteroplasmy load, and 83.4 (IQR = 9.6) and 41.6 (IQR = 4.5) among participants with high microheteroplasmy load, respectively. Reproduced by kind permission of UK Biobank ©

After stratification by frailty status, higher mtDNAcn was significantly associated with lower hazards of all-cause dementia, AD, and non-AD dementia among pre-frail/frail participants only, and these associations were not significant among non-frail participants (Table 2, Fig. 1). After stratification by a median split of microheteroplasmy load, mtDNA was significantly associated with non-AD dementia risk among participants with higher microheteroplasmy load, and these associations were not significant among those with lower microheteroplasmy load (Table 2, Fig. 2). Results remained similar when analyses were repeated in those aged 60 years or older (Supplementary Table 2) and additional adjustment for comorbidities (Supplementary Table 3). Among those with higher microheteroplasmies, associations between higher mtDNAcn and lower hazard of non-AD dementia remained significant after further adjustment for physical activity (HR = 0.880, 95% CI: 0.801 to 0.966, *p* = 0.007).

### mtDNA copy number and cognitive function

**Fig. 3:**
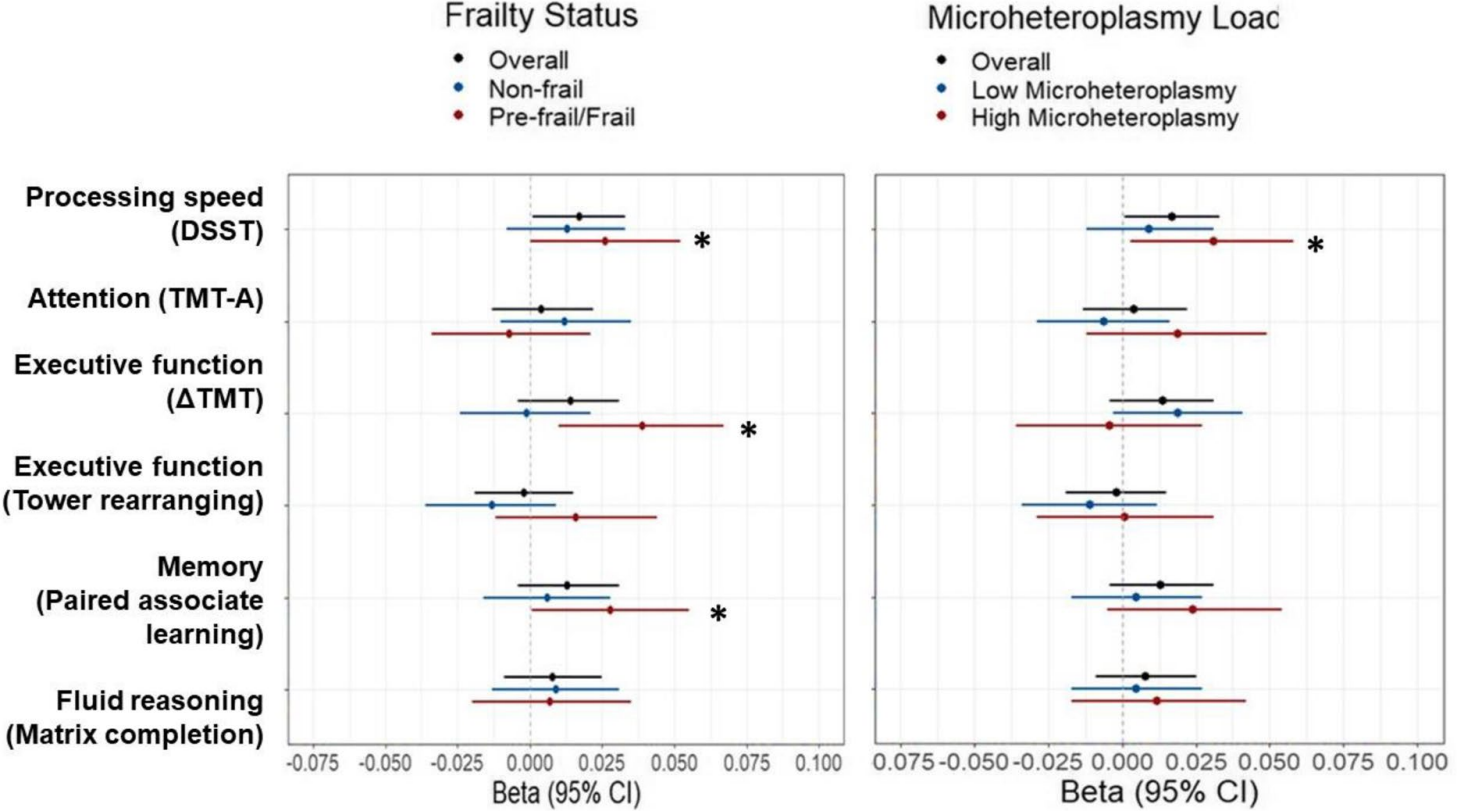
Dot plots of the overall and stratified associations between mitochondrial DNA copy number and cognitive measures. Legend: DSST digit symbol substitution test, TMT trail making test. The sign of TMT scores was flipped to be consistent with other cognitive measures; higher values indicate higher performance. Reproduced by kind permission of UK Biobank ©

Overall, more mtDNAcn was significantly associated with higher DSST (*p* = 0.036) but not with other cognitive measures (Table 3, Fig. 3). After stratification by frailty status or by a median split of microheteroplasmies, mtDNAcn was only significantly associated with DSST in those who were pre-frail/frail or those who had higher microheteroplasmies (*p* = 0.048 and 0.029, respectively). In addition, mtDNAcn was also significantly associated with delta TMT and paired associate learning (*p* = 0.007 and 0.045, respectively) in those who were pre-frail/frail, and not in non-frail participants (Table 3, Fig. 3).

**Table 3:** Associations between mitochondrial DNA copy number and cognitive measures in the overall sample and stratified by frailty status and microheteroplasmy load

		Overall sample	Stratify by frailty status		Stratify by microheteroplasmies	
			Non-frail	Pre-frail/frail	Lower levels	Higher levels
	*N*	*β* (95% CI), *p* value				
DSST (processing speed)	13,938	**0.017** **(0.001, 0.033)** **0.036**	0.013 (− 0.008, 0.033) 0.234	**0.026** **(0.0003, 0.052)** **0.048**	0.009 (− 0.012, 0.031) 0.388	**0.031** **(0.003, 0.058)** **0.029**
TMT part A (attention)	13,532	− 0.004 (− 0.022, 0.013) 0.638	− 0.012 (− 0.035, 0.010) 0.282	0.007 (− 0.021, 0.034) 0.625	0.006 (− 0.016, 0.029) 0.575	− 0.019 (− 0.049, 0.012) 0.236
Delta TMT (executive function)	13,426	− 0.014 (− 0.031, 0.004) 0.127	0.001 (− 0.021, 0.024) 0.899	− **0.039** **(**− **0.067,** − **0.010)** **0.007**	− 0.019 (− 0.041, 0.003) 0.096	0.004(− 0.027, 0.036) 0.794
Tower rearranging (executive function)	13,817	− 0.002 (− 0.019, 0.015) 0.827	− 0.013 (− 0.036, 0.009) 0.238	0.016 (− 0.012, 0.044) 0.255	− 0.011 (− 0.034, 0.012) 0.338	0.001 (− 0.029, 0.031) 0.925
Paired associate learning (memory)	14,092	0.013 (− 0.004, 0.031) 0.120	0.006 (− 0.016, 0.028) 0.598	**0.028** **(0.0006, 0.055)** **0.045**	0.005 (− 0.017, 0.027) 0.668	0.024 (− 0.005, 0.054) 0.106
Matrix patten completion (fluid reasoning)	13,933	0.008 (− 0.009, 0.025) 0.379	0.009 (− 0.013, 0.031) 0.433	0.007 (− 0.020, 0.035) 0.590	0.005 (− 0.017, 0.027) 0.673	0.012 (− 0.017, 0.042) 0.404

Notes: mtDNAcn and cognitive measures were standardized to *Z* scores

*Delta TMT* = TMT part B − part A

Higher values of the TMT part A and delta TMT indicate lower performance. The bold number reflects significant associations at two-tailed *p* < 0.05. Microheteroplasmies are split into lower and higher levels by a median value

*DSST* digit symbol substitution test, *TMT* trail making test

## Discussion

This large population-based study of mtDNAcn assessed from WGS establishes three important findings. First, higher mtDNAcn is associated with a reduced risk of dementia, especially non-AD dementia, independent of demographics and *APOE ε4* carrier status. Second, the strength and significance of the association between mtDNAcn and dementia are stronger among participants who were pre-frail/frail or had higher levels of microheteroplasmy, which are both phenotypes indicating a high level of cell damage and reduced resilience. Third, higher mtDNAcn is associated with higher specific cognitive measures. The association with cognitive function is also stronger in those who were pre-frail/frail or had higher microheteroplasmy load.

Findings of our study point to a role of mitochondrial dysfunction in non-AD dementia risk but not in AD dementia, which is consistent with previous findings of mtDNAcn estimated from exome [10]. It is worth noting that recent data have shown that blood-based mtDNAcn is associated with AD pathology including amyloid and tau from post-mortem brain tissues in older persons who died between 81 and 89 years old [27]. The fact that we did not observe a relationship with AD risk in our study may be because our participants were in their mid-50 s at the time of mtDNAcn assessment and the age of AD diagnosis ranged from 49 to 83 years old. However, contrary to this hypothesis our results remained substantially unchanged in a sensitivity analysis restricted to participants who were 60 years or older. The adjudication of AD diagnosis, which was based on hospital or GP records only, may have resulted in misclassification of the outcome.

We expanded on the previous report that the risk of dementia is particularly high in frail persons by showing that the association between mtDNAcn and dementia is stronger in individuals who are frail and have a high load of mtDNA microheteroplasmy in circulating cells. These findings are consistent with our initial hypothesis that unstable and fast damage accumulation, operationalized here as frailty and level of mtDNA microheteroplasmy, affects the role of mitochondrial mass and function in maintaining brain health. Several lines of research have shown that frailty is a state of biological instability and a high predisposition to rapid health deterioration, functional decline, and high mortality risk. Since mitochondrial function provides the energy that fuels many of the resilience mechanisms that contrast biological damage accumulation, such as DNA repair, proteostasis, and autophagy, it is not surprising that mitochondrial mass appears to be protective against dementia, especially in those who had evidence of more severe damage accumulation.

We found a consistent effect of the presence of high microheteroplasmy load on the relationship between mtDNAcn and non-AD dementia, which further confirms and strengthens the effect of frailty. The stronger associations under frailty or high somatic mutations lay on the hypothesis that mtDNA heteroplasmy load represents a status of mitochondrial genomic instability and that somatic mutation accumulation in mtDNA is a proxy measure for this status. mtDNA mutation may be caused by the direct effect of ROS or other stresses compounded with insufficient DNA repair. Increased mitochondrial biogenesis may lead to high mtDNA replication and mitochondrial biogenesis and increase mitochondrial mass to provide the energy required for multiple biological resilience mechanisms. Hence, higher mtDNAcn appears a protective factor against dementia, especially in the context of mitochondrial stress. These findings are consistent with our previous findings of an interaction effect between mtDNAcn and heteroplasmy load on skeletal muscle oxidative capacity in community-dwelling adults. In particular, mtDNAcn was shown to be positively associated with skeletal muscle mitochondrial function in those with higher levels of heteroplasmy load [28]. Studies demonstrated that mitochondrial OXPHOS is affected when mtDNA mutations exceed a certain threshold and both mtDNAcn and mutations are important determinants of disease conditions [29]. Collectively, these findings suggest that it is critical to consider mitochondrial DNA damage or mutant phenotype when examining the association between mtDNAcn and health outcomes. Notably, we did not find a direct relationship between microheteroplasmies and dementia risk which was in line with previous data that heteroplasmies were not associated with brain pathologies [11]. One recent study examines mtDNA variation and cognitive function, but it is limited to 16 mitochondrial-related regions [30]. Future studies are warranted to examine whether specific allelic mutations in mtDNA are implicated in the development of all-cause dementia and subtypes.

Findings from the cognitive outcomes extended prior research by examining multiple cognitive domains and tested the effects of somatic mutations and frailty. A recent meta-analysis study reported that mtDNAcn was associated with cognitive composite scores but did not test specific domains or measures [31]. We investigated cognitive domains, including memory, executive function, attention, and fluid reasoning. mtDNAcn showed an overall association with processing speed measured by DSST. Importantly, the relationship between mtDNAcn with processing speed was prominent in those who were pre-frail or frail or those with high somatic mutations. Further, among people who were pre-frail or frail, mtDNAcn also showed an association with memory and executive function. These findings are in line with previous findings on the relationship between mtDNAcn and cognition and provide additional insights into which cognitive domains that mtDNAcn is strongly associated with and the effect of biological damage on this relationship. Future studies with sufficient longitudinal follow-ups should explore the relationship between mtDNAcn and cognitive change over time.

This study has several strengths. First, this is the largest population-based study of mtDNAcn estimated from WGS data which is considered the most accurate estimation of mtDNAcn to date [32]. Notably, WGS data from the blood can include mitochondrial derived vesicles (MDVs) that carry mtDNA and age may affect the secretion of MDVs. However, the secretion of MDVs may unlikely affect the mtDNAcn estimation because MDVs primarily transport damaged mtDNA in response to mitochondrial damage or stress, and only a small fraction of total mtDNAcn may be carried by MDVs [33]. Our findings were independent of age, and results remained similar when the sample was restricted to 60 years or older. Second, stratification by indices of biological damage operationalized by frailty or higher microheteroplasmies advances our understanding of the effect of biological damage on the relationship between mtDNAcn and dementia risk. Third, microheteroplasmies are carefully identified using a previously suggested threshold which is likely to reflect somatic mutations. This study has limitations. Participants of this study are relatively younger at the time of mtDNAcn assessment compared to other aging studies more suitable to study dementia etiology. Second, UK Biobank routine healthcare data can be used for all-cause dementia, but the positive predictive value for AD dementia and vascular dementia is lower which may have underestimated the potential relationship with specific types of dementia [34].

In conclusion, among community-dwelling adults, mtDNAcn predicts future dementia, especially non-AD dementia. The relationship is pronounced under the condition of frailty or high mitochondrial damage. Individuals with high mitochondrial damage or frailty may be important subgroups for early prevention and intervention to prevent or delay the onset of dementia. Future studies are warranted to investigate underlying mechanisms.

## Supplementary Information

Below is the link to the electronic supplementary material.

Supplementary file1 (DOCX 50 KB)

## Data Availability

Data analyzed in this study are available upon request by proposal submission via the BLSA website portal (https://www.blsa.nih.gov/how-apply). All requests to access the BLSA datasets are reviewed by the BLSA Data Sharing Proposal Review Committee and are also subject to approval from the NIH Institutional Review Board.

## Declarations

### Data statement

Some of these findings were presented as posters at the Alzheimer’s Association International Conference Annual meetings in 2023 (Amsterdam, Netherlands) and 2024 (Philadelphia, PA, USA).
