# Supplementary material for: Mitochondrial DNA copy number associated dementia risk by somatic mutations and frailty
Source: GeroScience. 2024 Sep 23;47(1):825–35. doi: 10.1007/s11357-024-01355-1 (PMC11872790; doi:10.1007/s11357-024-01355-1)
Supplement: Supplementary file 1 — Supplementary file1 (DOCX 50 KB) [file 11357_2024_1355_MOESM1_ESM.docx]

**Supplementary Table 1: Diagnostic codes used to identify dementia cases.**

| **Dementia phenotype** | **Vocabulary** | **Code** | **Description** |
| --- | --- | --- | --- |
| All-cause dementia (ACD) | ICD10 | F00 | Dementia in Alzheimer disease |
| All-cause dementia (ACD) | ICD10 | F01 | Vascular dementia |
| All-cause dementia (ACD) | ICD10 | F02 | Dementia in other diseases classified elsewhere |
| All-cause dementia (ACD) | ICD10 | F03 | Unspecified dementia |
| All-cause dementia (ACD) | ICD10 | G30 | Alzheimer disease |
| All-cause dementia (ACD) | ICD10 | A81.0 | Creutzfeldt-Jakob disease |
| All-cause dementia (ACD) | ICD10 | F05.1 | Delirium superimposed on dementia |
| All-cause dementia (ACD) | ICD10 | F10.6 | Mental and behavioural disorders due to use of alcohol |
| All-cause dementia (ACD) | ICD10 | G31.0 | Circumscribed brain atrophy |
| All-cause dementia (ACD) | ICD10 | G31.1 | Senile degeneration of brain, not elsewhere classified |
| All-cause dementia (ACD) | ICD10 | G31.8 | Other specified degenerative diseases of nervous system |
| All-cause dementia (ACD) | ICD10 | I67.3 | Progressive vascular leukoencephalopathy / Binswanger's disease |
| All-cause dementia (ACD) | Read2 | A410. | Kuru |
| All-cause dementia (ACD) | Read2 | A411. | Jakob-Creutzfeldt disease |
| All-cause dementia (ACD) | Read2 | A4110 | Sporadic Creutzfeldt-Jakob disease |
| All-cause dementia (ACD) | Read2 | A4110 | Sporadic CJD (Creutzfeldt-Jakob disease) |
| All-cause dementia (ACD) | Read2 | E00.. | Senile and presenile organic psychotic conditions |
| All-cause dementia (ACD) | Read2 | E00.. | Senile dementia |
| All-cause dementia (ACD) | Read2 | E00.. | Senile/presenile dementia |
| All-cause dementia (ACD) | Read2 | E000. | Uncomplicated senile dementia |
| All-cause dementia (ACD) | Read2 | E001. | Presenile dementia |
| All-cause dementia (ACD) | Read2 | E0010 | Uncomplicated presenile dementia |
| All-cause dementia (ACD) | Read2 | E0011 | Presenile dementia with delirium |
| All-cause dementia (ACD) | Read2 | E0012 | Presenile dementia with paranoia |
| All-cause dementia (ACD) | Read2 | E0013 | Presenile dementia with depression |
| All-cause dementia (ACD) | Read2 | E001z | Presenile dementia NOS |
| All-cause dementia (ACD) | Read2 | E002. | Senile dementia with depressive or paranoid features |
| All-cause dementia (ACD) | Read2 | E0020 | Senile dementia with paranoia |
| All-cause dementia (ACD) | Read2 | E0021 | Senile dementia with depression |
| All-cause dementia (ACD) | Read2 | E002z | Senile dementia with depressive or paranoid features NOS |
| All-cause dementia (ACD) | Read2 | E003. | Senile dementia with delirium |
| All-cause dementia (ACD) | Read2 | E004. | Arteriosclerotic dementia |
| All-cause dementia (ACD) | Read2 | E004. | Multi infarct dementia |
| All-cause dementia (ACD) | Read2 | E0040 | Uncomplicated arteriosclerotic dementia |
| All-cause dementia (ACD) | Read2 | E0041 | Arteriosclerotic dementia with delirium |
| All-cause dementia (ACD) | Read2 | E0042 | Arteriosclerotic dementia with paranoia |
| All-cause dementia (ACD) | Read2 | E0043 | Arteriosclerotic dementia with depression |
| All-cause dementia (ACD) | Read2 | E004z | Arteriosclerotic dementia NOS |
| All-cause dementia (ACD) | Read2 | E00y. | Other senile and presenile organic psychoses |
| All-cause dementia (ACD) | Read2 | E041. | Dementia in conditions EC |
| All-cause dementia (ACD) | Read2 | Eu00. | [X]Dementia in Alzheimer's disease |
| All-cause dementia (ACD) | Read2 | Eu000 | [X]Dementia in Alzheimer's disease with early onset |
| All-cause dementia (ACD) | Read2 | Eu000 | [X]Presenile dementia,Alzheimer's type |
| All-cause dementia (ACD) | Read2 | Eu000 | [X] Primary degenerative dementia of Alzheimer's type, presenile onset |
| All-cause dementia (ACD) | Read2 | Eu000 | [X]Alzheimer's disease type 2 |
| All-cause dementia (ACD) | Read2 | Eu001 | [X]Dementia in Alzheimer's disease with late onset |
| All-cause dementia (ACD) | Read2 | Eu001 | [X]Alzheimer's disease type 1 |
| All-cause dementia (ACD) | Read2 | Eu001 | [X]Senile dementia,Alzheimer's type |
| All-cause dementia (ACD) | Read2 | Eu001 | [X]Primary degenerative dementia of Alzheimer's type, senile onset |
| All-cause dementia (ACD) | Read2 | Eu002 | [X]Dementia in Alzheimer's dis, atypical or mixed type |
| All-cause dementia (ACD) | Read2 | Eu00z | [X]Dementia in Alzheimer's disease, unspecified |
| All-cause dementia (ACD) | Read2 | Eu00z | [X]Alzheimer's dementia unspec |
| All-cause dementia (ACD) | Read2 | Eu01. | [X]Vascular dementia |
| All-cause dementia (ACD) | Read2 | Eu01. | [X]Arteriosclerotic dementia |
| All-cause dementia (ACD) | Read2 | Eu010 | [X]Vascular dementia of acute onset |
| All-cause dementia (ACD) | Read2 | Eu011 | [X]Multi-infarct dementia |
| All-cause dementia (ACD) | Read2 | Eu011 | [X]Predominantly cortical dementia |
| All-cause dementia (ACD) | Read2 | Eu012 | [X]Subcortical vascular dementia |
| All-cause dementia (ACD) | Read2 | Eu013 | [X]Mixed cortical and subcortical vascular dementia |
| All-cause dementia (ACD) | Read2 | Eu01y | [X]Other vascular dementia |
| All-cause dementia (ACD) | Read2 | Eu01z | [X]Vascular dementia, unspecified |
| All-cause dementia (ACD) | Read2 | Eu02. | [X]Dementia in other diseases classified elsewhere |
| All-cause dementia (ACD) | Read2 | Eu020 | [X]Dementia in Pick's disease |
| All-cause dementia (ACD) | Read2 | Eu021 | [X]Dementia in Creutzfeldt-Jakob disease |
| All-cause dementia (ACD) | Read2 | Eu022 | [X]Dementia in Huntington's disease |
| All-cause dementia (ACD) | Read2 | Eu023 | [X]Dementia in Parkinson's disease |
| All-cause dementia (ACD) | Read2 | Eu025 | [X]Lewy body dementia |
| All-cause dementia (ACD) | Read2 | Eu02y | [X]Dementia in other specified diseases classif elsewhere |
| All-cause dementia (ACD) | Read2 | Eu02z | [X] Unspecified dementia |
| All-cause dementia (ACD) | Read2 | Eu02z | [X] Presenile dementia NOS |
| All-cause dementia (ACD) | Read2 | Eu02z | [X] Presenile psychosis NOS |
| All-cause dementia (ACD) | Read2 | Eu02z | [X] Primary degenerative dementia NOS |
| All-cause dementia (ACD) | Read2 | Eu02z | [X] Senile dementia NOS |
| All-cause dementia (ACD) | Read2 | Eu02z | [X] Senile psychosis NOS |
| All-cause dementia (ACD) | Read2 | Eu02z | [X] Senile dementia, depressed or paranoid type |
| All-cause dementia (ACD) | Read2 | F110. | Alzheimer's disease |
| All-cause dementia (ACD) | Read2 | F1100 | Alzheimer's disease with early onset |
| All-cause dementia (ACD) | Read2 | F1101 | Alzheimer's disease with late onset |
| All-cause dementia (ACD) | Read2 | F111. | Pick's disease |
| All-cause dementia (ACD) | Read2 | F112. | Senile degeneration of brain |
| All-cause dementia (ACD) | Read2 | F116. | Lewy body disease |
| All-cause dementia (ACD) | Read2 | F118. | Frontotemporal degeneration |
| All-cause dementia (ACD) | Read2 | F11x7 | Cerebral degeneration due to Jakob - Creutzfeldt disease |
| All-cause dementia (ACD) | Read2 | F21y2 | Binswanger's disease |
| All-cause dementia (ACD) | Read2 | F21y2 | Binswanger's encephalopathy |
| All-cause dementia (ACD) | Read2 | Fyu30 | [X]Other Alzheimer's disease |
| All-cause dementia (ACD) | CTV3 | A410. | Kuru |
| All-cause dementia (ACD) | CTV3 | A410. | Kuru encephalitis |
| All-cause dementia (ACD) | CTV3 | A411. | Creutzfeldt-Jakob disease |
| All-cause dementia (ACD) | CTV3 | A411. | CJD - Creutzfeldt-Jakob disease |
| All-cause dementia (ACD) | CTV3 | A411. | JCD - Jakob-Creutzfeldt disease |
| All-cause dementia (ACD) | CTV3 | A411. | Jakob-Creutzfeldt disease |
| All-cause dementia (ACD) | CTV3 | E000. | Uncomplicated senile dementia |
| All-cause dementia (ACD) | CTV3 | E001. | Presenile dementia |
| All-cause dementia (ACD) | CTV3 | E0010 | Uncomplicated presenile dementia |
| All-cause dementia (ACD) | CTV3 | E0011 | Presenile dementia with delirium |
| All-cause dementia (ACD) | CTV3 | E0012 | Presenile dementia with paranoia |
| All-cause dementia (ACD) | CTV3 | E0013 | Presenile dementia with depression |
| All-cause dementia (ACD) | CTV3 | E001z | Presenile dementia NOS |
| All-cause dementia (ACD) | CTV3 | E002. | Senile dementia with depressive or paranoid features |
| All-cause dementia (ACD) | CTV3 | E0020 | Senile dementia with paranoia |
| All-cause dementia (ACD) | CTV3 | E0021 | Senile dementia with depression |
| All-cause dementia (ACD) | CTV3 | E002z | Senile dementia with depressive or paranoid features NOS |
| All-cause dementia (ACD) | CTV3 | E003. | Senile dementia with delirium |
| All-cause dementia (ACD) | CTV3 | E004. | Arteriosclerotic dementia (including [multi infarct dementia]) |
| All-cause dementia (ACD) | CTV3 | E004. | Arteriosclerotic dementia |
| All-cause dementia (ACD) | CTV3 | E004. | Multi infarct dementia |
| All-cause dementia (ACD) | CTV3 | E0040 | Uncomplicated arteriosclerotic dementia |
| All-cause dementia (ACD) | CTV3 | E0041 | Arteriosclerotic dementia with delirium |
| All-cause dementia (ACD) | CTV3 | E0042 | Arteriosclerotic dementia with paranoia |
| All-cause dementia (ACD) | CTV3 | E0043 | Arteriosclerotic dementia with depression |
| All-cause dementia (ACD) | CTV3 | E004z | Arteriosclerotic dementia NOS |
| All-cause dementia (ACD) | CTV3 | E00z. | Senile or presenile psychoses NOS |
| All-cause dementia (ACD) | CTV3 | E00z. | [X]Presenile psychosis NOS |
| All-cause dementia (ACD) | CTV3 | E041. | Dementia in conditions EC |
| All-cause dementia (ACD) | CTV3 | Eu00. | [X]Dementia in Alzheimer's disease |
| All-cause dementia (ACD) | CTV3 | Eu00. | Dementia in Alzheimer's disease |
| All-cause dementia (ACD) | CTV3 | Eu00. | DAT - Dementia Alzheimer's type |
| All-cause dementia (ACD) | CTV3 | Eu002 | [X]Dementia in Alzheimer's dis, atypical or mixed type |
| All-cause dementia (ACD) | CTV3 | Eu00z | [X]Dementia in Alzheimer's disease, unspecified |
| All-cause dementia (ACD) | CTV3 | Eu011 | [X]Dementia: [multi-infarct] or [predominantly cortical] |
| All-cause dementia (ACD) | CTV3 | Eu011 | [X]Multi-infarct dementia |
| All-cause dementia (ACD) | CTV3 | Eu011 | [X]Predominantly cortical dementia |
| All-cause dementia (ACD) | CTV3 | Eu01y | [X]Other vascular dementia |
| All-cause dementia (ACD) | CTV3 | Eu01z | [X]Vascular dementia, unspecified |
| All-cause dementia (ACD) | CTV3 | Eu02. | [X]Dementia in other diseases classified elsewhere |
| All-cause dementia (ACD) | CTV3 | Eu020 | [X]Dementia in Pick's disease |
| All-cause dementia (ACD) | CTV3 | Eu021 | [X]Dementia in Creutzfeldt-Jakob disease |
| All-cause dementia (ACD) | CTV3 | Eu022 | [X]Dementia in Huntington's disease |
| All-cause dementia (ACD) | CTV3 | Eu023 | [X]Dementia in Parkinson's disease |
| All-cause dementia (ACD) | CTV3 | Eu02y | [X]Dementia in other specified diseases classif elsewhere |
| All-cause dementia (ACD) | CTV3 | Eu041 | [X]Delirium superimposed on dementia |
| All-cause dementia (ACD) | CTV3 | F110. | Alzheimer's disease |
| All-cause dementia (ACD) | CTV3 | F110. | AD - Alzheimer's disease |
| All-cause dementia (ACD) | CTV3 | F111. | Pick's disease |
| All-cause dementia (ACD) | CTV3 | F112. | Senile degeneration of brain |
| All-cause dementia (ACD) | CTV3 | F11x7 | Cerebral degeneration due to Creutzfeldt-Jakob disease |
| All-cause dementia (ACD) | CTV3 | F21y2 | Binswanger's disease |
| All-cause dementia (ACD) | CTV3 | F21y2 | Binswanger's encephalopathy |
| All-cause dementia (ACD) | CTV3 | F21y2 | Subcortical arteriosclerotic encephalopathy |
| All-cause dementia (ACD) | CTV3 | F21y2 | Subcortical atherosclerotic dementia |
| All-cause dementia (ACD) | CTV3 | F21y2 | Chronic progressive subcortical encephalopathy |
| All-cause dementia (ACD) | CTV3 | Fyu30 | [X]Other Alzheimer's disease |
| All-cause dementia (ACD) | CTV3 | X002w | Dementia |
| All-cause dementia (ACD) | CTV3 | X002x | Dementia in Alzheimer's disease with early onset |
| All-cause dementia (ACD) | CTV3 | X002x | Dementia in Alzheimer's disease - type 2 |
| All-cause dementia (ACD) | CTV3 | X002x | Presenile dementia, Alzheimer's type |
| All-cause dementia (ACD) | CTV3 | X002y | Familial Alzheimer's disease of early onset |
| All-cause dementia (ACD) | CTV3 | X002z | Non-familial Alzheimer's disease of early onset |
| All-cause dementia (ACD) | CTV3 | X0030 | Dementia in Alzheimer's disease with late onset |
| All-cause dementia (ACD) | CTV3 | X0030 | Dementia in Alzheimer's disease - type 1 |
| All-cause dementia (ACD) | CTV3 | X0030 | SDAT - Senile dementia, Alzheimer's type |
| All-cause dementia (ACD) | CTV3 | X0031 | Familial Alzheimer's disease of late onset |
| All-cause dementia (ACD) | CTV3 | X0032 | Non-familial Alzheimer's disease of late onset |
| All-cause dementia (ACD) | CTV3 | X0033 | Focal Alzheimer's disease |
| All-cause dementia (ACD) | CTV3 | X0034 | Frontotemporal dementia |
| All-cause dementia (ACD) | CTV3 | X0035 | Pick's disease with Pick bodies |
| All-cause dementia (ACD) | CTV3 | X0036 | Pick's disease with Pick cells and no Pick bodies |
| All-cause dementia (ACD) | CTV3 | X0037 | Frontotemporal degeneration |
| All-cause dementia (ACD) | CTV3 | X0037 | Lobar atrophy |
| All-cause dementia (ACD) | CTV3 | X0039 | Frontal lobe degeneration with motor neurone disease |
| All-cause dementia (ACD) | CTV3 | X003A | Lewy body disease |
| All-cause dementia (ACD) | CTV3 | X003A | LBD - Lewy body disease |
| All-cause dementia (ACD) | CTV3 | X003A | SDLT - Senile dementia of the Lewy body type |
| All-cause dementia (ACD) | CTV3 | X003A | Cortical Lewy body disease |
| All-cause dementia (ACD) | CTV3 | X003A | CLBD - Cortical Lewy body disease |
| All-cause dementia (ACD) | CTV3 | X003A | Diffuse Lewy body disease |
| All-cause dementia (ACD) | CTV3 | X003A | DLBD - Diffuse Lewy body disease |
| All-cause dementia (ACD) | CTV3 | X003A | Dementia of the Lewy body type |
| All-cause dementia (ACD) | CTV3 | X003G | Progressive aphasia in Alzheimer's disease |
| All-cause dementia (ACD) | CTV3 | X003H | Argyrophilic brain disease |
| All-cause dementia (ACD) | CTV3 | X003I | Post-traumatic dementia |
| All-cause dementia (ACD) | CTV3 | X003J | Punch drunk syndrome |
| All-cause dementia (ACD) | CTV3 | X003J | Dementia pugilistica |
| All-cause dementia (ACD) | CTV3 | X003R | Vascular dementia of acute onset |
| All-cause dementia (ACD) | CTV3 | X003T | Subcortical vascular dementia |
| All-cause dementia (ACD) | CTV3 | X003V | Mixed cortical and subcortical vascular dementia |
| All-cause dementia (ACD) | CTV3 | X003W | Semantic dementia |
| All-cause dementia (ACD) | CTV3 | X003X | Patchy dementia |
| All-cause dementia (ACD) | CTV3 | X003Y | Epileptic dementia |
| All-cause dementia (ACD) | CTV3 | X00R2 | Senile dementia |
| All-cause dementia (ACD) | CTV3 | X00R2 | SD - Senile dementia |
| All-cause dementia (ACD) | CTV3 | XE1aG | Dementia (& [presenile] or [senile]) |
| All-cause dementia (ACD) | CTV3 | XE1aG | Senile/presenile dementia |
| All-cause dementia (ACD) | CTV3 | XE1aG | Senile dementia |
| All-cause dementia (ACD) | CTV3 | XE1aG | Senile and presenile dementias |
| All-cause dementia (ACD) | CTV3 | XE1aG | Senile/presenile dement |
| All-cause dementia (ACD) | CTV3 | XE1Xs | Vascular dementia |
| All-cause dementia (ACD) | CTV3 | XE1Xs | Arteriosclerotic dementia |
| All-cause dementia (ACD) | CTV3 | XE1Xs | VAD - Vascular dementia |
| All-cause dementia (ACD) | CTV3 | XE1Xt | Other senile and presenile organic psychoses |
| All-cause dementia (ACD) | CTV3 | XE1Z6 | [X]Unspecified dementia |
| All-cause dementia (ACD) | CTV3 | Y00dJ | MID - Multi-infarct dementia |
| All-cause dementia (ACD) | CTV3 | Y00dM | Binswanger's encephalopathy |
| All-cause dementia (ACD) | CTV3 | Y00Gv | Dementia |
| All-cause dementia (ACD) | CTV3 | Y00Gw | Alzheimer's disease |
| All-cause dementia (ACD) | CTV3 | Y00H0 | Dementia in Alzheimer's disease with early onset |
| All-cause dementia (ACD) | CTV3 | Y00H2 | Presenile dementia, Alzheimer's type |
| All-cause dementia (ACD) | CTV3 | Y00H5 | Dementia in Alzheimer's disease with late onset |
| All-cause dementia (ACD) | CTV3 | Y00H7 | SDAT - Senile dementia, Alzheimer's type |
| All-cause dementia (ACD) | CTV3 | Y00HC | Pick's disease |
| All-cause dementia (ACD) | CTV3 | Y00Hk | Kuru |
| All-cause dementia (ACD) | CTV3 | Y00HL | Lewy body disease |
| All-cause dementia (ACD) | CTV3 | Y00Hv | Vascular dementia |
| All-cause dementia (ACD) | CTV3 | Y00Hw | Arteriosclerotic dementia |
| All-cause dementia (ACD) | CTV3 | Y00Hx | Vascular dementia of acute onset |
| All-cause dementia (ACD) | CTV3 | Y00Hz | Subcortical vascular dementia |
| All-cause dementia (ACD) | CTV3 | Y00I0 | Binswanger's disease |
| All-cause dementia (ACD) | CTV3 | Y00I1 | Mixed cortical and subcortical vascular dementia |
| All-cause dementia (ACD) | CTV3 | Y00La | Senile degeneration of brain |
| All-cause dementia (ACD) | CTV3 | Y016a | Senile dementia with depressive or paranoid features |
| All-cause dementia (ACD) | CTV3 | Y016b | Senile dementia with paranoia |
| All-cause dementia (ACD) | CTV3 | Y016c | Senile dementia with depression |
| All-cause dementia (ACD) | CTV3 | Y016d | Senile dementia with depressive or paranoid features NOS |
| All-cause dementia (ACD) | CTV3 | Y016e | Uncomplicated senile dementia |
| All-cause dementia (ACD) | CTV3 | Y016f | Dementia in conditions EC |
| All-cause dementia (ACD) | CTV3 | Y016M | Senile and presenile organic psychotic conditions |
| All-cause dementia (ACD) | CTV3 | Y016N | Senile or presenile psychoses NOS |
| All-cause dementia (ACD) | CTV3 | Y016O | Other senile and presenile organic psychoses |
| All-cause dementia (ACD) | CTV3 | Y016P | Presbyophrenic psychosis |
| All-cause dementia (ACD) | CTV3 | Y016S | Presenile dementia |
| All-cause dementia (ACD) | CTV3 | Y016T | Uncomplicated presenile dementia |
| All-cause dementia (ACD) | CTV3 | Y016U | Presenile dementia with delirium |
| All-cause dementia (ACD) | CTV3 | Y016V | Presenile dementia with paranoia |
| All-cause dementia (ACD) | CTV3 | Y016w | Arteriosclerotic dementia with paranoia |
| All-cause dementia (ACD) | CTV3 | Y016W | Presenile dementia with depression |
| All-cause dementia (ACD) | CTV3 | Y016x | Arteriosclerotic dementia with depression |
| All-cause dementia (ACD) | CTV3 | Y016X | Presenile dementia NOS |
| All-cause dementia (ACD) | CTV3 | Y016y | Arteriosclerotic dementia NOS |
| All-cause dementia (ACD) | CTV3 | Y016Y | Senile dementia |
| All-cause dementia (ACD) | CTV3 | Y016z | Arteriosclerotic dementia with delirium |
| All-cause dementia (ACD) | CTV3 | Y016Z | Senile dementia with delirium |
| All-cause dementia (ACD) | CTV3 | Y0170 | Uncomplicated arteriosclerotic dementia |
| All-cause dementia (ACD) | CTV3 | Ya1Sy | Frontotemporal degeneration |
| All-cause dementia (ACD) | CTV3 | Ya2nk | Cerebral degeneration presenting primarily with dementia |
| All-cause dementia (ACD) | CTV3 | YadT0 | Multi-infarct dementia |
| All-cause dementia (ACD) | CTV3 | Yady4 | Cerebral degeneration due to Creutzfeldt-Jakob disease |
| All-cause dementia (ACD) | CTV3 | Yao4I | [X]Lewy body dementia |
| All-cause dementia (ACD) | CTV3 | YaxJd | Sporadic Creutzfeldt-Jakob disease |
| All-cause dementia (ACD) | CTV3 | YaxJe | Sporadic CJD (Creutzfeldt-Jakob disease) |
| All-cause dementia (ACD) | CTV3 | YMAhP | Alzheimer's disease with early onset |
| All-cause dementia (ACD) | CTV3 | YMAhQ | Alzheimer's disease with late onset |
| All-cause dementia (ACD) | CTV3 | YMB0i | [X]Dementia in Alzheimer's disease |
| All-cause dementia (ACD) | CTV3 | YMB0l | [X]Dementia in Alzheimer's dis, atypical or mixed type |
| All-cause dementia (ACD) | CTV3 | YMB0m | [X]Dementia in Alzheimer's disease, unspecified |
| All-cause dementia (ACD) | CTV3 | YMB0s | [X]Other vascular dementia |
| All-cause dementia (ACD) | CTV3 | YMB0t | [X]Vascular dementia, unspecified |
| All-cause dementia (ACD) | CTV3 | YMB0u | [X]Dementia in other diseases classified elsewhere |
| All-cause dementia (ACD) | CTV3 | YMB0v | [X]Dementia in Pick's disease |
| All-cause dementia (ACD) | CTV3 | YMB0w | [X]Dementia in Creutzfeldt-Jakob disease |
| All-cause dementia (ACD) | CTV3 | YMB0x | [X]Dementia in Huntington's disease |
| All-cause dementia (ACD) | CTV3 | YMB0y | [X]Dementia in Parkinson's disease |
| All-cause dementia (ACD) | CTV3 | YMB10 | [X]Dementia in other specified diseases classif elsewhere |
| All-cause dementia (ACD) | CTV3 | YMB11 | [X]Unspecified dementia |
| All-cause dementia (ACD) | CTV3 | YMB15 | [X]Delirium superimposed on dementia |
| All-cause dementia (ACD) | CTV3 | YMB1Z | [X]Mental and behavioural disorders due to use of alcohol: residual and late-onset psychotic disorder |
| All-cause dementia (ACD) | CTV3 | YMBwl | [X]Presenile psychosis NOS |
| All-cause dementia (ACD) | CTV3 | YMJwc | Jakob-Creutzfeldt disease |
| All-cause dementia (ACD) | CTV3 | YMK1N | [X] Primary degenerative dementia NOS |
| All-cause dementia (ACD) | CTV3 | YMK1O | [X] Senile dementia NOS |
| All-cause dementia (ACD) | CTV3 | YMK1P | [X] Senile psychosis NOS |
| Alzheimer's disease (AD) | ICD10 | F00 | Dementia in Alzheimer disease |
| Alzheimer's disease (AD) | ICD10 | G30 | Alzheimer disease |
| Alzheimer's disease (AD) | Read2 | Eu00. | [X]Dementia in Alzheimer's disease |
| Alzheimer's disease (AD) | Read2 | Eu000 | [X]Dementia in Alzheimer's disease with early onset |
| Alzheimer's disease (AD) | Read2 | Eu000 | [X]Presenile dementia,Alzheimer's type |
| Alzheimer's disease (AD) | Read2 | Eu000 | [X] Primary degenerative dementia of Alzheimer's type, presenile onset |
| Alzheimer's disease (AD) | Read2 | Eu000 | [X]Alzheimer's disease type 2 |
| Alzheimer's disease (AD) | Read2 | Eu001 | [X]Dementia in Alzheimer's disease with late onset |
| Alzheimer's disease (AD) | Read2 | Eu001 | [X]Alzheimer's disease type 1 |
| Alzheimer's disease (AD) | Read2 | Eu001 | [X]Senile dementia,Alzheimer's type |
| Alzheimer's disease (AD) | Read2 | Eu001 | [X]Primary degenerative dementia of Alzheimer's type, senile onset |
| Alzheimer's disease (AD) | Read2 | Eu002 | [X]Dementia in Alzheimer's dis, atypical or mixed type |
| Alzheimer's disease (AD) | Read2 | Eu00z | [X]Dementia in Alzheimer's disease, unspecified |
| Alzheimer's disease (AD) | Read2 | Eu00z | [X]Alzheimer's dementia unspec |
| Alzheimer's disease (AD) | Read2 | F110. | Alzheimer's disease |
| Alzheimer's disease (AD) | Read2 | F1100 | Alzheimer's disease with early onset |
| Alzheimer's disease (AD) | Read2 | F1101 | Alzheimer's disease with late onset |
| Alzheimer's disease (AD) | Read2 | Fyu30 | [X]Other Alzheimer's disease |
| Alzheimer's disease (AD) | CTV3 | Eu00. | [X]Dementia in Alzheimer's disease |
| Alzheimer's disease (AD) | CTV3 | Eu00. | Dementia in Alzheimer's disease |
| Alzheimer's disease (AD) | CTV3 | Eu00. | DAT - Dementia Alzheimer's type |
| Alzheimer's disease (AD) | CTV3 | Eu002 | [X]Dementia in Alzheimer's dis, atypical or mixed type |
| Alzheimer's disease (AD) | CTV3 | Eu00z | [X]Dementia in Alzheimer's disease, unspecified |
| Alzheimer's disease (AD) | CTV3 | F110. | Alzheimer's disease |
| Alzheimer's disease (AD) | CTV3 | F110. | AD - Alzheimer's disease |
| Alzheimer's disease (AD) | CTV3 | Fyu30 | [X]Other Alzheimer's disease |
| Alzheimer's disease (AD) | CTV3 | X002x | Dementia in Alzheimer's disease with early onset |
| Alzheimer's disease (AD) | CTV3 | X002x | Dementia in Alzheimer's disease - type 2 |
| Alzheimer's disease (AD) | CTV3 | X002x | Presenile dementia, Alzheimer's type |
| Alzheimer's disease (AD) | CTV3 | X002y | Familial Alzheimer's disease of early onset |
| Alzheimer's disease (AD) | CTV3 | X002z | Non-familial Alzheimer's disease of early onset |
| Alzheimer's disease (AD) | CTV3 | X0030 | Dementia in Alzheimer's disease with late onset |
| Alzheimer's disease (AD) | CTV3 | X0030 | Dementia in Alzheimer's disease - type 1 |
| Alzheimer's disease (AD) | CTV3 | X0030 | SDAT - Senile dementia, Alzheimer's type |
| Alzheimer's disease (AD) | CTV3 | X0031 | Familial Alzheimer's disease of late onset |
| Alzheimer's disease (AD) | CTV3 | X0032 | Non-familial Alzheimer's disease of late onset |
| Alzheimer's disease (AD) | CTV3 | X0033 | Focal Alzheimer's disease |
| Alzheimer's disease (AD) | CTV3 | X003G | Progressive aphasia in Alzheimer's disease |
| Alzheimer's disease (AD) | CTV3 | Y00Gw | Alzheimer's disease |
| Alzheimer's disease (AD) | CTV3 | Y00H0 | Dementia in Alzheimer's disease with early onset |
| Alzheimer's disease (AD) | CTV3 | Y00H2 | Presenile dementia, Alzheimer's type |
| Alzheimer's disease (AD) | CTV3 | Y00H5 | Dementia in Alzheimer's disease with late onset |
| Alzheimer's disease (AD) | CTV3 | Y00H7 | SDAT - Senile dementia, Alzheimer's type |
| Alzheimer's disease (AD) | CTV3 | YMAhP | Alzheimer's disease with early onset |
| Alzheimer's disease (AD) | CTV3 | YMAhQ | Alzheimer's disease with late onset |
| Alzheimer's disease (AD) | CTV3 | YMB0i | [X]Dementia in Alzheimer's disease |
| Alzheimer's disease (AD) | CTV3 | YMB0l | [X]Dementia in Alzheimer's dis, atypical or mixed type |
| Alzheimer's disease (AD) | CTV3 | YMB0m | [X]Dementia in Alzheimer's disease, unspecified |

**Supplementary Table 2. Associations between mtDNAcn and dementia risk and interaction effects with frailty status and microheteroplasmy load in those 60 and older**

|  |  | **All-cause dementia** | **AD dementia** | **Non-AD dementia** |
| --- | --- | --- | --- | --- |
|  |  | **HR (95% CI), p-value** | | |
| Overall | mtDNAcn | 0.955  (0.916, 0.996)  0.032 | 0.980  (0.922, 1.04)  0.511 | 0.930  (0.877, 0.986)  0.016 |
| Frailty interaction | mtDNAcn | 1.01  (0.950, 1.07)  0.753 | 1.04  (0.961, 1.14)  0.304 | 0.966  (0.882, 1.06)  0.449 |
|  | Frailty | 1.49  (1.38, 1.61)  <0.001 | 1.24  (1.11, 1.38)  <0.001 | 1.80  (1.62, 2.00)  <0.001 |
|  | mtDNAcn-by-Frailty | 0.911  (0.842, 0.986)  0.020 | 0.887  (0.793, 0.992)  0.036 | 0.946  (0.846, 1.06)  0.336 |
| Micro-heteroplasmy interaction | mtDNAcn | 0.950  (0.908, 0.994)  0.027 | 0.985  (0.922, 1.05)  0.642 | 0.919  (0.863, 0.978)  0.008 |
|  | Microheteroplasmy load | 1.00  (0.962, 1.04)  0.960 | 1.00  (0.948, 1.06)  0.915 | 0.996  (0.943, 1.05)  0.896 |
|  | mtDNAcn-by-microheteroplasmy | 0.972  (0.932, 1.01)  0.180 | 1.02  (0.956, 1.08)  0.584 | 0.934  (0.881, 0.989)  0.019 |

Notes: mtDNAcn was standardized as Z score. All models were adjusted for age, age-squared term (if significant), sex, race and ethnicity, education, assessment center, smoking status, *APOE ε4* carrier status, autosomal DNA sequencing coverage white blood cell count, lymphocytes percentage. Bold number reflects significant associations at two-tailed p<0.05.

**Supplementary Table 3. Associations between mtDNAcn and dementia risk and interaction effects with frailty status and microheteroplasmy load after additional adjustment for comorbidities**

|  |  | **All-cause dementia** | **AD dementia** | **Non-AD dementia** |
| --- | --- | --- | --- | --- |
|  |  | **HR (95% CI), p-value** | | |
| Overall | mtDNAcn | 0.959  (0.922, 0.996)  0.032 | 0.977  (0.923, 1.03)  0.427 | 0.941  (0.893, 0.992)  0.025 |
| Frailty interaction | mtDNAcn | 0.999  (0.944, 1.06)  0.967 | 1.03  (0.953, 1.11)  0.454 | 0.963  (0.887, 1.05)  0.370 |
|  | Frailty | 1.42  (1.33, 1.53)  <0.001 | 1.19  (1.07, 1.31)  <0.001 | 1.69  (1.53, 1.86)  <0.001 |
|  | mtDNAcn-by-Frailty | 0.935  (0.869, 1.00)  0.067 | 0.906  (0.815, 1.01)  0.065 | 0.968  (0.875, 1.07)  0.529 |
| Micro-heteroplasmy interaction | mtDNAcn | 0.954  (0.915, 0.994)  0.026 | 0.986  (0.927, 1.05)  0.654 | 0.925  (0.874, 0.980)  0.008 |
|  | Microheteroplasmy load | 1.00  (0.967, 1.04)  0.869 | 1.02  (0.964, 1.07)  0.535 | 0.989  (0.940, 1.04)  0.663 |
|  | mtDNAcn-by-microheteroplasmy | 0.971  (0.934, 1.01)  0.140 | 1.02  (0.959, 1.08)  0.593 | 0.936  (0.888, 0.986)  0.012 |

Notes: same as notes in the supplementary Table 2.
